# Impact of clinical pharmacist-led pharmacotherapy on long-term outcomes in cardiovascular in-patients: A three-year cohort study: Retraction

**DOI:** 10.1097/MD.0000000000047473

**Published:** 2026-01-16

**Authors:** Hui Chen, Chao Wang, Xueqin He, Ting Jiang, Xin Wan

The article, “Impact of clinical pharmacist-led pharmacotherapy on long-term outcomes in cardiovascular in-patients: A three-year cohort study,”^[1]^ which appears in Volume 104, Issue 44 of *Medicine*, is being retracted due to the authors’ unlicensed use of The Morisky Medication Adherence Scale, otherwise known as the Morisky Scale (MMAS-8). The authors were contacted and given an opportunity to obtain a retroactive license but did not reply to requests from Dr. Morisky’s legal team or the publisher. All authors were notified of this retraction.
